# Will Smartphone Applications Replace the Insertable Cardiac Monitor in the Detection of Atrial Fibrillation? The First Comparison in a Case Report of a Cryptogenic Stroke Patient

**DOI:** 10.3389/fcvm.2022.839853

**Published:** 2022-03-23

**Authors:** Femke Wouters, Henri Gruwez, Julie Vranken, Ludovic Ernon, Dieter Mesotten, Pieter Vandervoort, David Verhaert

**Affiliations:** ^1^Limburg Clinical Research Center/Mobile Health Unit, Faculty of Medicine and Life Sciences, Hasselt University, Hasselt, Belgium; ^2^Department Future Health, Ziekenhuis Oost-Limburg, Genk, Belgium; ^3^Department of Cardiology, Ziekenhuis Oost-Limburg, Genk, Belgium; ^4^Department of Cardiovascular Sciences, University of Leuven, Leuven, Belgium; ^5^Department of Neurology, Ziekenhuis Oost-Limburg, Genk, Belgium; ^6^Department of Anesthesiology, Ziekenhuis Oost-Limburg, Genk, Belgium

**Keywords:** atrial fibrillation, cryptogenic stroke, mobile health, case report, insertable cardiac monitor (ICM)

## Abstract

**Background and Case:**

This case report exemplifies the clinical application of non-invasive photoplethysmography (PPG)-based rhythm monitoring in the awakening mobile health (mHealth) era to detect symptomatic and asymptomatic paroxysmal atrial fibrillation (AF) in a cryptogenic stroke patient. Despite extensive diagnostic workup, the etiology remains unknown in one out of three ischemic strokes (i.e., cryptogenic stroke). Prolonged cardiac monitoring can reveal asymptomatic atrial fibrillation in up to one-third of this population. This case report describes a cryptogenic stroke patient who received prolonged cardiac monitoring with an insertable cardiac monitor (ICM) as standard of care. In the context of a clinical study, the patient simultaneously monitored his heart rhythm with a PPG-based smartphone application. AF was detected simultaneously on both the ICM and smartphone application after three days of monitoring. Similar AF burden was detected during follow-up (five episodes, median duration of 28 and 34 h on ICM and mHealth, respectively, *p* = 0.5). The detection prompted the initiation of oral anticoagulation and AF catheter ablation procedure.

**Conclusion:**

This is the first report of the cryptogenic stroke patient in whom PPG-based mHealth was able to detect occurrence and burden of the symptomatic and asymptomatic paroxysmal AF episodes with similar precision as ICM. It accentuates the potential role of PPG-based mHealth in prolonged cardiac rhythm monitoring in cryptogenic stroke patients.

## Introduction

The etiology of stroke remains unknown in one-third of all ischemic stroke patients despite extensive diagnostic workup (i.e., cryptogenic stroke) (1). Prolonged cardiac monitoring can reveal the often asymptomatic atrial fibrillation (AF) in 12–33% patients using insertable cardiac monitors (ICMs) (2, 3). These patients are almost three times as likely to suffer from a recurrent stroke as non-AF-related stroke patients (4). Since oral anticoagulation can only be initiated after AF diagnosis, this has significant implications for secondary prevention (5).

According to the European Society of Cardiology (ESC) guidelines, prolonged cardiac monitoring is recommended in cryptogenic stroke and transient ischemic attack (TIA) patients since it increases the detection rate of AF by a factor of six (2, 5). The ongoing REMOTE study on cryptogenic stroke patients with implanted ICMs [approved by the medical ethics committees (Ziekenhuis Oost-Limburg, Genk, Belgium and Hasselt University, Hasselt, Belgium): 19/0093U, ClinicalTrials.gov Identifier: NCT05006105] investigates the added value of photoplethysmography (PPG)-based mobile health (mHealth) in AF detection using spot-check and semi-continuous measurements on the smartphone or smartwatch, respectively. The FibriCheck® application (Qompium NV, Hasselt, Belgium) was used as a tool in this study. This app has both CE mark and FDA approval. It is qualified to detect AF in patients with medical-grade precision (sensitivity and specificity are 96 and 97%, respectively) (6, 7). This case report presents the detection of symptomatic and asymptomatic, paroxysmal AF episodes with a minimum duration of approximately 19 h in a cryptogenic stroke patient enrolled in the REMOTE study using an mHealth smartphone application.

## Case Description

A 59-year old male with a past medical history of arterial hypertension, a sedentary lifestyle, and who was a former smoker woke up with aphasia and headache. His medication regimen consisted of chlortalidone 50 mg, quinapril 20 mg, and atenolol 25 mg. He presented to the emergency department the next day with normal vital parameters and word-finding difficulties, resulting in a National Institutes of Health Stroke Scale (NIHSS) on admission of one.

### Diagnostic Assessment

Initial labs showed no electrolyte or metabolic disturbances; glycemia was 129 mg%. The electrocardiogram (ECG) was normal except mild sinus bradycardia of 52 bpm. A computed tomography (CT) scan of the brain indicated recent ischemia in the left temporoparietal cortex. The CT angiography of the carotid arteries showed no cause of the stroke. Brain magnetic resonance imaging confirmed recent ischemia with diffusion restriction in the corticosubcortical posterior temporal area of 47 mm, with a stroke volume of 20 ml in the left middle cerebral artery area (M3). Both gray and white matter were affected. Another focal area of diffusion restriction was distinguished in the paramedian right occipital lobe. There was no hemorrhagic transformation. The EEG did not demonstrate signs of epilepsy.

The transesophageal echocardiogram could not confirm a cardiac source of emboli. Cardiac monitoring during 67 h on the stroke unit could not detect AF. HbA1c was normal, 5.7%. The LDL cholesterol level was 79 mg/dl; total cholesterol was 145 mg/dl. The thrombophilia screening panel, including anti-cardiolipin and lupus anticoagulant, was negative. The patient was sent home on dual antiplatelet therapy with acetylsalicylic acid 80 mg and clopidogrel 75 mg during three weeks and was told to continue only clopidogrel 75 mg after that. Furthermore, atorvastatin 40 mg was initiated. After hospital discharge, a 24-h blood pressure monitor showed no hypertension. A seven-day ECG Holter showed no AF episodes or pauses. However, 82 bradycardia events, 235 ventricular ectopic beats, and 481 supraventricular ectopic beats were detected.

### Long-Term Cardiac Monitoring

The stroke was finally defined as cryptogenic due to a negative seven-day ECG Holter. As a result, an ICM was indicated for prolonged cardiac rhythm monitoring to detect asymptomatic AF. Furthermore, the patient was included in the clinical double-blind REMOTE study in which ICMs are compared to PPG-based mHealth on either a smartphone or smartwatch. An ICM was inserted seven weeks after the stroke to monitor the heart rhythm continuously until battery end-of-service (i.e., average duration of three years). The patient was randomized to the smartphone monitoring group and was asked to perform two one-minute spot-checks using the FibriCheck® application each day, and additional spot-checks could be performed in case of symptoms during a period of 6 months. This application uses the PPG signal, which is interpreted and classified by an algorithm. After an offline validation, the result was available for the researcher, yet blinded for both patient and caregiver.

### Detection of Atrial Fibrillation

The use of mHealth was initiated on the day of ICM insertion. The time to first AF detection was three days. This first AF episode with rapid ventricular response was detected on both ICM and mHealth. The ICM reported an AF episode lasting 28 h. During this period, five mHealth spot-checks were performed. All of them were identified as AF; only one of these episodes was reported to be symptomatic. The initially cryptogenic stroke was now considered to be caused by cardioembolism due to AF. Since this first AF episode occurred during the weekend, it took five days before the cardiologist switched from antiplatelet to anticoagulation therapy based on the ICM data (i.e., mHealth was blinded for both patient and caregiver). Six weeks after ICM insertion, bisoprolol 5 mg was initiated.

Due to the patient’s young age, the absence of structural cardiac disorders, and the paroxysmal nature of this AF, an ablation procedure was performed to isolate the pulmonary veins. After the ablation, flecainide 100 mg was initiated. No AF was detected in the six weeks following the ablation, resulting in the cessation of flecainide 100 mg and bisoprolol 5 mg. He continued to use edoxaban 60 mg, chlortalidone 25 mg, atorvastatin 40 mg, and quinapril 20 mg. Neither mHealth nor ICM detected AF within 8 months after ablation.

### Comparison Between Insertable Cardiac Monitor and Photoplethysmography- Based Mobile Health

**FIGURE 1:**
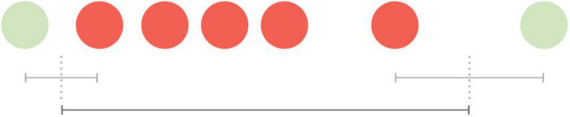
Visualization of the estimated duration of an atrial fibrillation episode measured by spot-checks. The time between the last green spot-check (no AF) and the first red spot-check (AF) divided by two, plus the time between the first and last red spot-check, plus the time between the last red spot-check and the first green spot-check divided by two.

Before the ablation procedure, five AF episodes were detected on both the ICM and mHealth application. The durations of these AF episodes are presented in Table 1. The AF duration detected by the mHealth application was estimated as follows (Figure 1); the time between the last green spot-check (i.e., no AF) and the first red spot-check (i.e., AF) divided by two, plus the time between the first and last red spot-check, plus the time between the last red spot-check and the first green spot-check divided by two. The duration of the AF episodes thus calculated with mHealth (*mdn* = 34 h 19 min), was not significantly different from the ICM-registered episodes (*mdn* = 28 h 22 min, *Z* = –0.674, *p* = 0.5). The AF burden was calculated as the proportion of time the patient was in AF during a monitoring period (8). The overall AF burden between ICM insertion until ablation was 8% according to the ICM and 9% based on mHealth.

**TABLE 1:** Duration of the AF episodes based on ICM and PPG-based mHealth.

AF episode number	AF duration (ICM)	AF duration (mHealth)	*p*-value
1	28 h 22 min	36 h 18 min	
2	28 h 44 min	41 h 24 min	
3	26 h 14 min	26 h 46 min	
4	20 h 4 min	18 h 51 min	
5	37 h 14 min	34 h 19 min	
Total AF duration	140 h 38 min	157 h 38 min	
Median [IQR] AF duration	28 h 22 min [23 h 9 min–32 h 59 min]	34 h 19 min [22 h 49 min–38 h 51 min]	0.5

*AF, atrial fibrillation; ICM, insertable cardiac monitor; mHealth, mobile health. P-value was obtained via a Wilcoxon signed-rank test.*

Over a period of 180 days (i.e., 6 months), 230 measurements were performed using a smartphone. Simultaneously, the ICM collected 4,320 h of continuous data. Compliance was defined as the total number of spot-checks performed, divided by the total number of recommended spot-checks. Motivation was defined as the number of days with at least two daily spot-checks divided by the number of days. The compliance to the mHealth application prior to ablation was 88%, the motivation was 56%. After ablation, this decreased to a compliance of 46% and a motivation of 35%. Despite the moderate motivation of this patient to perform spot-checks, all AF episodes were detected by the mHealth application, with a similar AF burden as the continuous monitoring of an ICM. It is important to note that no other arrhythmias were detected using the mHealth application before or after the AF episodes. Furthermore, only three AF spot-checks (19%) were symptomatic (i.e., palpations and dizziness); two of these were recorded within the same hour.

**FIGURE 2:**
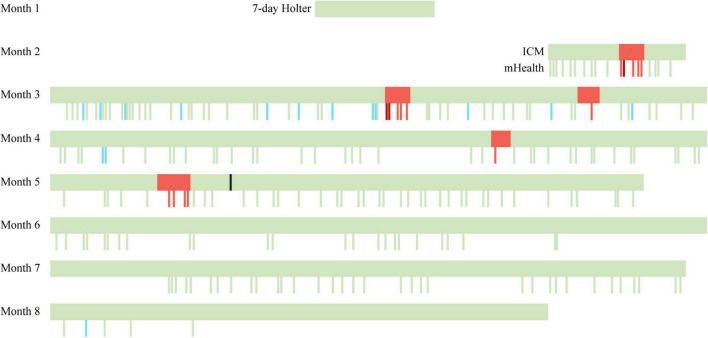
Timeline of continuous and spot-check measurements performed by ICM and mHealth. A seven-day ECG Holter was used in the first month. At the end of the second month, the ICM was inserted, and the use of PPG-based mHealth was initiated. Green bars indicate a sinus rhythm, red bars indicate an AF episode, dark red bars indicate a symptomatic AF episode as reported in the mHealth app, blue bars indicate insufficient data quality, and the black bar indicates the ablation. ICM, insertable cardiac monitor; mHealth, mobile health.

This patient was found to have primarily asymptomatic paroxysmal AF episodes after suffering a cryptogenic stroke. The AF episodes were concurrently detected by an ICM and a PPG-based mHealth smartphone application with an artificial intelligence algorithm to detect AF. The application detected all AF episodes identified by the ICM (Figure 2). Moreover, there were no false-positive mHealth recordings.

## Discussion

This case report discusses the first head-to-head comparison between continuous cardiac monitoring using an ICM and spot-check PPG-based rhythm monitoring in a cryptogenic stroke patient. The time until AF detection in this cryptogenic stroke patient using PPG-based mHealth was equal to the ICM, the gold standard for AF monitoring. Furthermore, despite a tremendous difference in data quantity, PPG-based mHealth was able to detect paroxysmal AF with a similar AF burden as the ICM in a cryptogenic stroke patient. During the third and fourth AF episodes, only one mHealth recording was performed. Nevertheless, the duration of these episodes was very similar between ICM and mHealth. However, the most accurate estimation of AF episode duration with mHealth is expected to be achieved when performing recordings frequently and regularly.

This case exemplifies a real-world clinical application of PPG-based rhythm monitoring in the awakening mHealth era. The detection of AF after cryptogenic stroke has a tremendous impact on treatment strategy and clinical outcomes. Therefore, the ESC recommends long-term cardiac monitoring using an ICM in cryptogenic stroke and TIA patients (5). Despite the clinical evidence of ICMs as demonstrated in the CRYSTAL-AF study and the recommendations in the guidelines, the use of long-term cardiac follow-up in these patients is not yet standard of care and thus underutilized (2, 9).

Compared to ICMs, PPG-based rhythm monitoring has some advantages. It is non-invasive, less expensive, and can be used anywhere, anytime (10, 11). Furthermore, it allows context and symptom reporting by the patient (12). Moreover, it identifies AF when it lasts at least 30 s of a 1-min recording, whereas multiple ICM devices require at least 2 min of AF (9, 13, 14). More PPG-based mHealth approaches are being developed, which can pave the path toward their use in cryptogenic stroke or TIA patients’ follow-up and secondary prevention (15).

In this case report, PPG-based mHealth was used on a smartphone by performing spot-check measurements. Consequently, longer AF episodes, similar to those detected in this patient, are more likely to be identified by two spot-check recordings per day. On the other hand, short AF episodes might have been missed when performing only two one-minute recordings in 24 h. Nevertheless, smartwatches can offer semi-continuous rhythm monitoring, approximating the continuous nature of ICMs (14). Further research is necessary to determine the duration of AF episodes that can be detected with spot-check or semi-continuous rhythm monitoring.

The Apple Heart Study, the Fitbit Heart Study, and the Huawei Heart Study already illustrated the potential of PPG-based rhythm monitoring using smartwatches to detect AF in a more general population. However, these studies used mHealth as a screening tool for primary prevention. As such, these studies were not performed in a cryptogenic stroke or TIA population. Moreover, a 24-h Holter or 7-day Holter was used to confirm AF. Therefore, there was only a limited time window where both PPG and ECG were used concurrently. This is in large contrast with ECG monitoring using an ICM with concurrent PPG monitoring for 6 months (16–18). In addition, spot-check recordings performed with a smartphone differ from semi-continuous rhythm monitoring performed with a smartwatch. When using a phone, the patient is stationary and aware that a measurement is being recorded. Moreover, these recordings can be performed when the patient experiences symptoms such as palpations. On the other hand, using a smartwatch, recordings are performed when the patient is performing its daily activities, resulting in data that is more prone to motion artifacts. Finally, another mHealth tool that detects AF but uses ECG instead of PPG is the AliveCor KardiaMobile. Compared with the PPG-based mHealth used in the REMOTE study, the AliveCor demonstrated equivalent diagnostic performance. However, a significant limitation of the hand-held ECG device is the necessity to purchase additional hardware (19).

### Study Limitations

A limitation in this case report is blinding the PPG-based mHealth results during the study. This has two consequences. First, if the results were unblinded, a recording that is suspicious for AF might prompt the patient to perform more recordings. This could improve the estimation of the AF episode duration. Second, no action nor time to action can be attributed to the detection of AF by the mHealth tool. Furthermore, the ESC guidelines state that when AF is detected by a screening tool such as mHealth, a confirmation of AF should be obtained using an ECG recording. Therefore, this confirmation is necessary to diagnose AF, and thus, to initiate anticoagulant therapy (5). However, it could be debated that in this high-risk population (i.e., secondary prevention of cryptogenic stroke patients), AF detected by PPG-based rhythm monitoring is sufficient to start therapy. However, more research is necessary to substantiate this decision. Secondly, this case report compares the detection of AF between mHealth and ICM in only one patient, limiting the extrapolation to the broader population. Therefore, the ongoing REMOTE study is essential to collect more data and provide more insight. Finally, short AF episodes may still be missed given the nature of intermittent monitoring using PPG-based mHealth. However, the clinical relevance of short AF episodes requires further investigation (8).

## Conclusion

This is the first report of the cryptogenic stroke patient in whom PPG-based mHealth was able to detect occurrence and burden of paroxysmal AF episodes with similar precision as ICM. ICM is the most performant rhythm monitoring device but is expensive, invasive, and currently underutilized. This case demonstrated the feasibility of implementing PPG-based mHealth monitoring as a low-cost and non-invasive tool. The potential role of PPG-based mHealth in prolonged cardiac rhythm monitoring in cryptogenic stroke patients should be validated in larger patient population.

### Patient Perspective

A questionnaire was conducted after using mHealth and indicated an equal sense of safety and reliability of both mHealth and ICM. Furthermore, the smartphone app was reported to be interesting, supportive, and easy to learn.

## Data Availability Statement

The raw data supporting the conclusions of this article will be made available by the authors, without undue reservation.

## Ethics Statement

The studies involving human participants were reviewed and approved by Comité Medische Ethiek, Ziekenhuis Oost-Limburg and Hasselt University. The patients/participants provided their written informed consent to participate in this study.

## Author Contributions

PV contributed to the diagnosis and treatment of this patient. FW collected the data, performed the statistical analysis, and drafted this manuscript. All authors read, reviewed, and edited the manuscript.

## Conflict of Interest

The authors declare that the research was conducted in the absence of any commercial or financial relationships that could be construed as a potential conflict of interest.

## Publisher’s Note

All claims expressed in this article are solely those of the authors and do not necessarily represent those of their affiliated organizations, or those of the publisher, the editors and the reviewers. Any product that may be evaluated in this article, or claim that may be made by its manufacturer, is not guaranteed or endorsed by the publisher.
